# Right ventricular size assessed by cardiovascular MRI may predict mortality after left ventricular assist device placement

**DOI:** 10.1186/1532-429X-17-S1-P181

**Published:** 2015-02-03

**Authors:** Mitchell Timmons, Aimee C Welsh, Dhiraj Baruah, Kaushik Shahir, Jason Rubenstein

**Affiliations:** Cardiovascular Medicine, Medical College of Wisconsin, Milwaukee, WI USA; Radiology, Medical College of Wisconsin, Milwaukee, WI USA

## Background

Early right ventricular (RV) failure after insertion of an implantable left ventricular assist device (LVAD) is associated with a poor prognosis and increased mortality. Improved assessment of RV volumes with cardiovascular magnetic resonance imaging (CMR) prior to LVAD placement may lead to more optimal patient selection.

## Methods

Patients were referred for cardiovascular magnetic resonance imaging prior to LVAD placement. We assessed the association of mortality to pre-LVAD right ventricular end systolic volume index (RVESVI), right ventricular end diastolic volume index (RVEDVI), left ventricular ejection fraction (LVEF) by CMR. Right ventricular stroke work index (RVSWI) was determined by pre-LVAD right heart catheterization.

## Results

**Figure 1:**
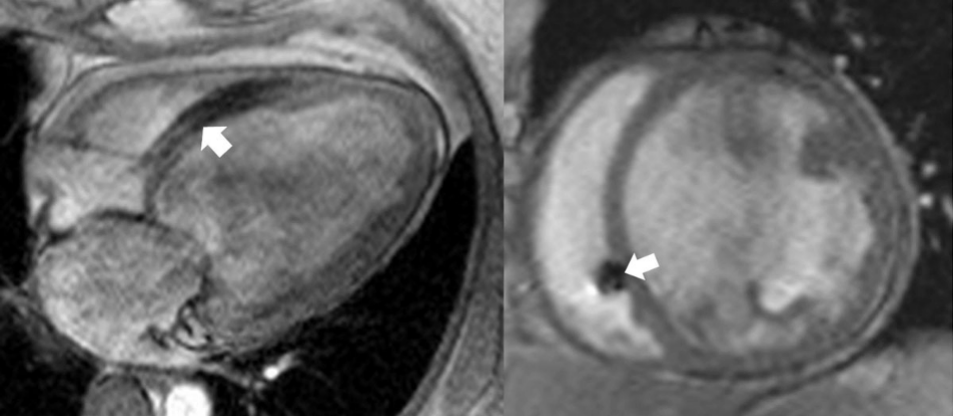
4-chamber (left) and short-axis (right) turbo-GRE images of right ventricle in pre-LVAD patient with ICD. Arrows indicate minimal ICD lead artifact.

We studied 15 consecutive patients (mean age, 54 ±12 years; 54% male) who received HVAD (60%) and HeartMate II (40%) LVADs for a diagnosis of dilated cardiomyopathy (57%) and ischemic cardiomyopathy (43%). 26.7% of patients had an implantable defibrillator (ICD) at the time of the MRI (figure 1), without any device-related complications or issues with image quality. A total of 3 deaths occurred during a median follow-up of 19 months (range, 3 to 31 months). By univariate analysis (table 1), mortality was significantly associated with increased RVESVI (116.9 ml ± 32.8 vs. 65.1 ml ± 25.6; p = 0.01) and RVEDVI (140.4 ml ± 21 vs. 86.6 ml ± 19.1; p = 0.001) when assessed prior to implantation of LVAD by CMR. There was no observed association in mortality with the more typical risk predictors of LVEF or RVSWI assessed prior to LVAD placement.

**Table 1:** Cardiovasuclar Magnetic Resonance imaging measurement of study population.

	Dead (n=3)	Alive (n=12)	p Value
RVEDVI (ml/ m^2^)	140.0±21.1	86.6±19.1	0.001
RVESVI (ml/ m^2^)	116.9±32.8	65.1±25.6	0.01
RVSV (ml)	45.8±42.8	40.8±23.9	0.784
RVEF (%)	17.1±15.1	27±18.9	0.42
Cl (ml/ min)	934.1±831.9	2770.9±1632.7	0.087
LVEF (%)	12.9±5.1	18.4±9.8	0.367
RVSWI (mmHg ml/m^2^)	342±151.7	441.7±132.1	0.275

## Conclusions

Increased RVESVI and RVEDVI assessed by CMR prior to implantation of LVADs is a predictor of post-implant mortality, while more typical measures such as LVEF was not. CMR acquisition and RV image quality was not hindered by implantable cardiac devices. Evaluation of RV volumes by CMR may improve risk-stratification and further refine patient selection for LVAD implantation.

## Funding

None.

